# Effects of Different Centrifugation Parameters on Equilibrium Solubility Measurements

**DOI:** 10.3390/mps8050116

**Published:** 2025-10-02

**Authors:** Rita Szolláth, Vivien Bárdos, Marcell Stifter-Mursits, Réka Angi, Károly Mazák

**Affiliations:** 1 Department of Pharmaceutical Chemistry, Semmelweis University, Hőgyes E. u. 9., H-1092 Budapest, Hungary; szollath.rita.mariann@semmelweis.hu (R.S.); bardos.vivien@semmelweis.hu (V.B.); stifter.marcell@phd.semmelweis.hu (M.S.-M.); angi.erzsebet@semmelweis.hu (R.A.); 2 Center for Pharmacology and Drug Research & Development, Semmelweis University, Üllői út 26, H-1085 Budapest, Hungary

**Keywords:** centrifugation, saturation shake-flask (SSF) method, equilibrium solubility, phase separation, progesterone, diclofenac sodium, hydrochlorothiazide, papaverine hydrochloride

## Abstract

The bioavailability of a drug is closely linked to its solubility, making its early determination essential in drug development. The saturation shake-flask (SSF) method is the gold standard protocol for this, which includes a phase separation step—either by sedimentation, filtration, or centrifugation. This step is critical, as it can directly influence the accuracy of the results. This study investigated the impact of centrifugation parameters—time and rotation speed—on solubility measurements. Additionally, we compared two sample preparation protocols: continuous stirring for 24 h versus 6 h of stirring followed by 18 h of sedimentation before centrifugation. Four model compounds were tested at three pH values using Britton–Robinson buffers. Centrifugation was conducted for 5, 10, or 20 min at either 5000 or 10,000 rpm. Results showed that pre-sedimented samples yielded solubility values closer to sedimentation-only references, while continuous stirring often led to overestimated values, particularly at higher speeds and longer durations. One such example was papaverine hydrochloride, that showed solubility values 60–70% higher than the reference after centrifugation at 10,000 rpm for 20 min without prior sedimentation. Lower standard deviations were observed with shorter, slower centrifugation, with 5 min and 5000 rpm yielding results closest to the reference values.

## 1. Introduction

Accurate and reproducible solubility data are crucial in pharmaceutical research and development [1,2,3,4]. Solubility not only informs early decisions around drug candidate selection but also directly influences formulation strategies, dosage form design, and predictions of in vivo performance [5,6,7,8,9,10]. Low solubility can lead to incomplete absorption of the administered drug. The solubility of active pharmaceutical ingredients (APIs) is a critical factor influencing their biopharmaceutical characteristics [11]. The saturation shake-flask (SSF) method is the gold standard for experimentally determining equilibrium solubility. Despite the emergence of several novel and automated methods, the SSF technique remains the benchmark for solubility determination due to its simplicity, broad applicability, and ability to reach true thermodynamic equilibrium [3,12,13,14,15]. The SSF method consists of five steps: (1) adding excess amount of solid to a solvent to create a suspension, (2) agitating the suspension at a controlled temperature until equilibrium is reached (3) separating the solid and liquid phase by sedimentation, filtration or centrifugation, (4) sampling and if necessary, dilution of the aliquots with the solvent, and (5) determination of the concentration of the compound using a suitable analytical method [16]. Several aspects of the SSF approach have been investigated [17], including the impact of excess solid, rotation speed, the effect of buffer composition, the pH-dependent solubility of ionizable compounds, the effect of biorelevant media, and the challenges associated with compounds exhibiting low dissolution rates [18,19,20,21,22,23,24,25,26,27,28,29,30,31]. Accurate solubility measurement depends not only on achieving equilibrium between the solid and the dissolved phases, but also on the effective and non-disruptive separation of these phases prior to analysis. In the SSF method, the choice of phase separation technique is of great importance, because it can disturb the heterogeneous equilibrium system or result in the inclusion of undissolved particles, compromising data integrity, resulting in misleading solubility values [32].

A study conducted by Baka et al. highlighted sedimentation as the most reliable and least intrusive method for phase separation in SSF experiments. Sedimentation allows the system to remain undisturbed over time, keeping the dynamic balance between solid and dissolved phases, making it the recommended first-choice approach for accurate equilibrium solubility determination [18]. Research by Monteiro et al. expanded upon the findings of Baka et al., establishing general conditions to be applied during the determination of equilibrium solubility [33].

However, the SSF method also has limitations. In practice, not all drug suspensions settle sufficiently through sedimentation alone, especially compounds that form aggregates, colloid systems, or small agglomerate particles, which can lead to turbidity or opalescence in the solution. In such cases, more active phase separation methods—centrifugation or filtration—are necessary to ensure that undissolved material is adequately removed [34].

By applying centrifugal force, suspended solids can be rapidly and effectively separated from the liquid phase. Unlike filtration, which carries risks of adsorption to filter materials, pore clogging, and material loss [16,35,36], centrifugation avoids direct contact with solid-phase barriers and is particularly useful when dealing with small-volume, heterogeneous systems. However, centrifugation is not without drawbacks [32]. If centrifugation parameters such as speed or duration are poorly optimized, they can disrupt the equilibrium by forcing smaller particles or colloids into the supernatant, falsely elevating the apparent solubility. On the other hand, inadequate centrifugation may fail to completely separate the phases causing inconsistencies during measurements.

Despite its critical role, the method of phase separation is often underreported in scientific literature. A previous review found the majority of SSF-based solubility studies did not specify how phase separation was performed, thus making the interpretation and reproducibility of solubility data more complicated. In the cases where the method was disclosed, filtration was the most commonly used technique (44%), followed by centrifugation (14%) and sedimentation (9%) [32].

While sedimentation is widely regarded as a low-intervention phase separation technique that helps preserve equilibrium conditions, its practical application is often limited due to slow kinetics and inefficiencies in settling—particularly for compounds with poor sedimentation properties. In such cases, centrifugation is typically employed to facilitate phase separation and obtain a clear supernatant for solubility analysis. However, despite its widespread use, the effect of centrifugation on equilibrium solubility values has not been systematically studied. Because of the potential impact of centrifugation on solubility results, understanding how centrifugation speed and separation time influence solubility values—especially in comparison to sedimentation—can help to obtain more accurate and replicable data. A recent study conducted about SSF measurements in viscous solvents highlighted how centrifugation speed influences the measured equilibrium solubility of „brick dust” compounds, causing overestimation [37]. With appropriate optimization, centrifugation can serve as a reliable and efficient method for phase separation, especially when sedimentation is insufficient. This is particularly important for low-solubility compounds, where small inaccuracies in measurement can have significant implications for formulation development and bioavailability predictions. This is of great importance, as nowadays, with the introduction of high-throughput screening systems, an increasing amount of drugs and new drug candidates are poorly soluble [11,38,39], belonging to the Biopharmaceutics Classification System (BCS) Class II or Class IV, both classes exhibiting low solubility, and in the case of Class IV compounds, low permeability as well [40].

Therefore, our aim was to perform a systematic evaluation of centrifugation parameters as part of the SSF method, comparing them with sedimentation-based measurements using four APIs across three pH values. Our objective was to assess whether centrifugation can yield solubility values comparable to those obtained by sedimentation and to identify the experimental conditions under which this is achievable. A deeper understanding of these parameters could also contribute to protocol optimization, reducing experimental duration while maintaining accuracy, thus aligning equilibrium solubility testing more closely with the demands of high-throughput drug discovery workflows.

## 2. Materials and Methods

### 2.1. Materials

**Figure 1:**
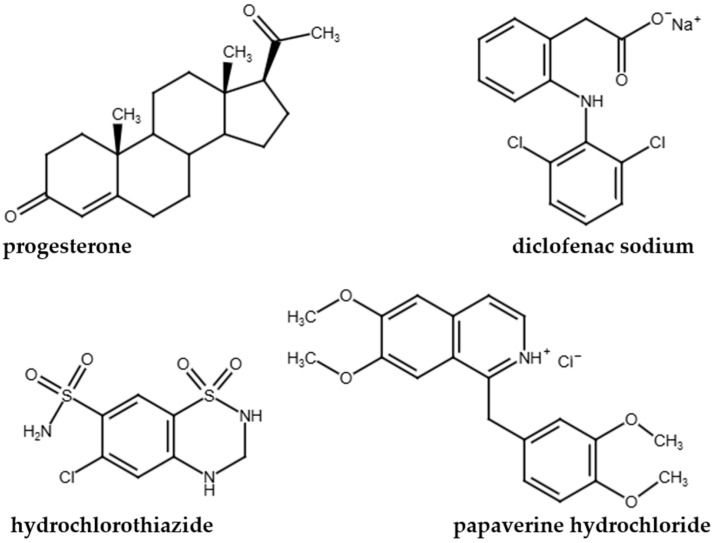
Structures of the model compounds.

The structure of the model compounds is shown in Figure 1, and their most important physico-chemical properties are presented in Table 1. Diclofenac sodium (DICL-NA), hydrochlorothiazide (HCT), papaverine hydrochloride (PAP-HCL) and progesterone (PROG) were purchased from Sigma-Aldrich Co. LLC. (St. Louis, MO, USA). The buffer components (acetic acid (99–100%), phosphoric acid (>85%), boric acid and sodium hydroxide) were provided by Molar Chemicals Ltd. (Halásztelek, Hungary). For the preparation of the buffers, pharmacopoeial grade distilled water was utilized.

### 2.2. Determination of Equilibrium Solubility Using Saturation Shake-Flask Method

To determine equilibrium solubility, the samples were added to Britton-Robinson buffer solutions (BRB) adjusted to specific pH values, obtaining a heterogenous system (Table S2). BR buffers were used in the experiments across a pH range of 2.0 to 11.0, prepared by combining 0.04 M acetic acid, 0.04 M phosphoric acid, and 0.04 M boric acid. The mixture was then titrated with varying amounts of 0.2 M NaOH to achieve the desired pH values. The ionic strength was kept constant (I = 0.15) across all pH values using appropriate amounts of inert KCl. Three pH values were typically used to evaluate each compound: one corresponding to the predominantly unionized form, one near the p*K*_a_ of the compound (where approximately 50% of the molecules are ionized), and one where the compound is fully ionized. The exception was progesterone, a neutral molecule, for which only a single pH condition was necessary. Notably, hydrochlorothiazide is a diprotic acid that exists as a dianion under sufficiently alkaline conditions. Ionizable compounds were specifically chosen due to the significant variation in solubility observed across different pH levels. This strategy enabled a more comprehensive assessment of how centrifugation parameters influence solubility. Different amounts of 0.2 M NaOH were used to adjust the pH of the buffer to the desired values, between pH 2.0 and 11.0.

Sample temperature was maintained at 25.0 ± 0.5 °C using a Lauda thermostat throughout the solubility measurements. Samples were stirred using the μDISS Profiler™ (Pion Inc., Billerica, MA, USA) at 150 rpm [33], the pH was checked after 1 h and adjusted, if necessary, with either 0.1 M HCl or 0.1 M NaOH. To confirm the reliability of the results, the pH was measured again at the end of each experiment. Prior to centrifugation, two different approaches were applied: stirring samples for 6 h, followed by 18 h of sedimentation, according to the standardized protocol [18], or continuously stirring samples for 24 h, without a subsequent sedimentation step.

**Figure 2:**
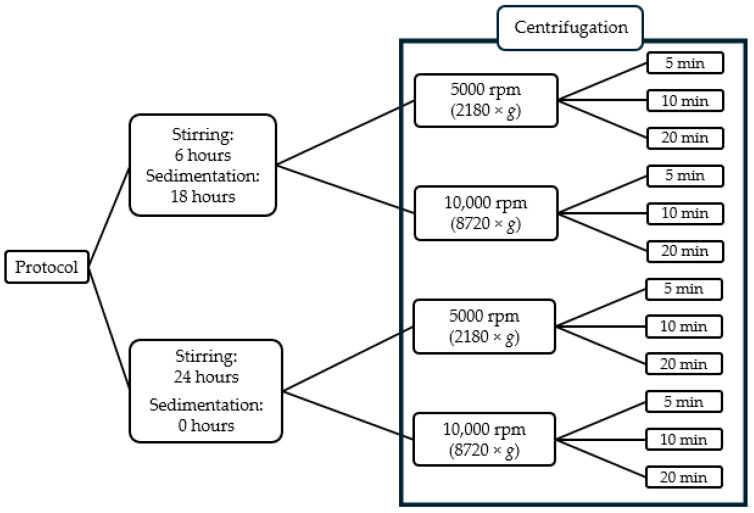
Combination of centrifugation parameters employed in the measurements.

From each sample, aliquots of 10 mL were transferred into centrifugation tubes and centrifuged using a Sartorius 2–16P type centrifuge. The samples were spun for the duration of either 5, 10 or 20 min at either 5000 rpm (corresponding to 2180× *g* relative centrifugal force) or 10,000 rpm (corresponding to 8720× *g*). Measurements were carried out at every combination of these parameters, the complete list of which is presented in Figure 2.

After centrifugation, the concentration was measured by the μDISS Profiler™ (Pion Inc., Billerica, MA, USA) equipped with UV probes. The aliquots were diluted with the buffer solution if necessary. The 2nd derivative method was utilized in the AuPRO™ 7.1 software (Pion Inc., Billerica, MA, USA) to determine the concentration of the samples after a calibration was conducted for each substance. Calibration was performed by adding small volumes of methanolic stock solutions with known concentrations to buffer solutions of defined pH. Separate calibration curves were prepared for each UV probe. For spectral evaluation, the second derivative method was applied to improve accuracy and reduce baseline effects. The resulting calibration parameters for each probe are provided in the Supplementary Information (Table S3).

At least three parallel measurements were carried out for each compound with each combination of variables (Table S1). Equilibrium solubility measurements using centrifugation were compared to those obtained by using only sedimentation.

### 2.3. Statistical Analysis

All solubility experiments were performed in replicates and are reported as mean values with the standard error of the mean (SEM). Normality of the solubility data was assessed visually by Q–Q plots, which did not indicate major deviations from normality (Figure S1). To assess whether centrifugation protocols differed significantly from the corresponding reference condition, a one-way analysis of variance (ANOVA) was conducted for each API–pH combination, followed by Dunnett’s post hoc test to compare each protocol against the reference (Figures S2–S11). This approach was selected to appropriately control multiple comparisons while maintaining statistical power. A significance threshold of *p* < 0.05 was applied. In addition, Welch’s unpaired two-tailed t-test was used to compare solubility values between sedimented and non-sedimented samples within each protocol (Figures S12–S21). This test accounts for unequal variances and differing sample sizes between groups. Three significant thresholds (*p* < 0.05, 0.01 and 0.001) were applied. Statistical analyses were performed in Python (version 3.12) using the scipy.stats module (SciPy version 1.11). Significant differences (*p* < 0.05) were marked with an asterisk in the solubility bar plots and in addition to that with red background in the summary table.

## 3. Results and Discussion

### 3.1. Determination of Reference Equilibrium Solubility Values

The gold-standard shake-flask method is routinely used in drug research and development. Its effective implementation relies heavily on the phase separating step. In our research, four drugs with different acid-base characteristics (see Table 1) were investigated by performing solubility measurements at various pH values: progesterone, diclofenac sodium, hydrochlorothiazide, and papaverine hydrochloride. The solubility measurements were conducted according to the standard protocol (stirring for 6 h and sedimenting for 18 h) and forgoing sedimentation completely and stirring our samples for 24 h instead. Afterwards, the effect of different centrifugation parameters was evaluated.

At first, we measured the equilibrium solubility using sedimentation after stirring as phase separation. We considered these as reference (100%) for comparison with the results obtained by centrifugation [18,32]. We selected these measurements because sedimentation is the least invasive phase separation method. It preserves the dynamic solubility equilibrium and minimizes disruption of the established solid–liquid balance. This technique allows undissolved particles to settle naturally, without the influence of external forces that could alter the concentration of the dissolved phase. These equilibrium solubility values are consistent with those measured previously by our research group using the same protocols [16]. Furthermore, the results from both studies are in good agreement with established literature data, supporting the reliability of our methods [27,41,42].

The measured reference solubility values are shown in Table 2.

The equilibrium solubility of progesterone was measured only at pH 7.4, as it is a neutral, lipophilic (log*P* = 3.48) molecule, thus its solubility is independent from pH.

Diclofenac is a highly lipophilic (log*P* = 4.51) monoprotic acid (p*K*_a_ = 3.99). For our measurements, diclofenac sodium was used, and we measured its solubility at three pH values at 25 °C. At pH 2.0, where the molecule is in its uncharged, neutral form (Equation (1)), the equilibrium solubility is the lowest. At pH 4.0, where around 50% of the molecule is already ionized (Equation (2)), the solubility of our compound slightly increased.

(1) $$\frac{\left[A^{-}\right]}{\left[HA\right]}=10^{2.0-3.99}=0.01$$

(2) $$\frac{\left[A^{-}\right]}{\left[HA\right]}=10^{4.0-3.99}=1.02$$

This indicates that at pH 2.0, only about 1% of the drug is ionized, while at pH 4.0, approximately 50% is ionized. Since the ionized form is significantly more soluble in aqueous media, this shift accounts for the observed increase in solubility.

At pH 10.0, where diclofenac is fully ionized, the solubility showed a 5670-fold increase compared to the unionized form.

Hydrochlorothiazide is a diprotic, weak acid with an endocyclic and an exocyclic sulfonamide group. Depending on the pH, the molecule exists as the neutral acid (H_2_A), monoanion (HA^−^), or dianion (A^2−^). Hydrochlorothiazide has a low lipophilicity, its log*P* value is close to 0. The solubility of hydrochlorothiazide was measured at three pH values at 25 °C. Using only sedimentation as phase separation, the solubility at pH 6.0 was found to be around 585 µg/mL, much higher than the more lipophilic diclofenac. At pH 8.8, where 45% of the molecule is unionized, 51% is monoanionic and 4% is present in dianionic form, the solubility increased by 50%. These values were derived from the mole fraction (*x*) of the various species, based on the compound’s known p*K*_a_ values and the corresponding buffer pH (Equations (3)–(6)). At pH 11.0, where most of hydrochlorothiazide is present in the dianionic form, the solubility is nearly hundred times higher than in acidic solutions.

(3) $${x(H}_{2}A)=\frac{{\left[H^{+}\right]}^{2}}{{\left[H^{+}\right]}^{2}+\left[H^{+}\right]K_{1}+K_{1}K_{2}}$$

(4) $$x({HA}^{-})=\frac{\left[H^{+}\right]K_{1}}{{\left[H^{+}\right]}^{2}+\left[H^{+}\right]K_{1}+K_{1}K_{2}}$$

(5) $$x(A^{2-})=\frac{K_{1}K_{2}}{{\left[H^{+}\right]}^{2}+\left[H^{+}\right]K_{1}+K_{1}K_{2}}$$

(6) $$K_{1}=[10^{-pK_{a1}}]\;K_{2}=[10^{-pK_{a2}}]$$

We used the hydrochloride salt of the monoprotic, lipophilic weak base papaverine. The equilibrium solubility was measured at three pH values. At pH 10.0, where the molecule is its neutral form, we measured its intrinsic solubility, which is around 17 µg/mL. At pH 6.4, 50% of the molecule is ionized, so the measured equilibrium concentration was slightly higher. The molecule is fully ionized at pH 3.0, therefore the solubility was the highest, around 2350 times higher than at pH 10.0.

### 3.2. The Effect of Centrifugation

**Figure 3:**
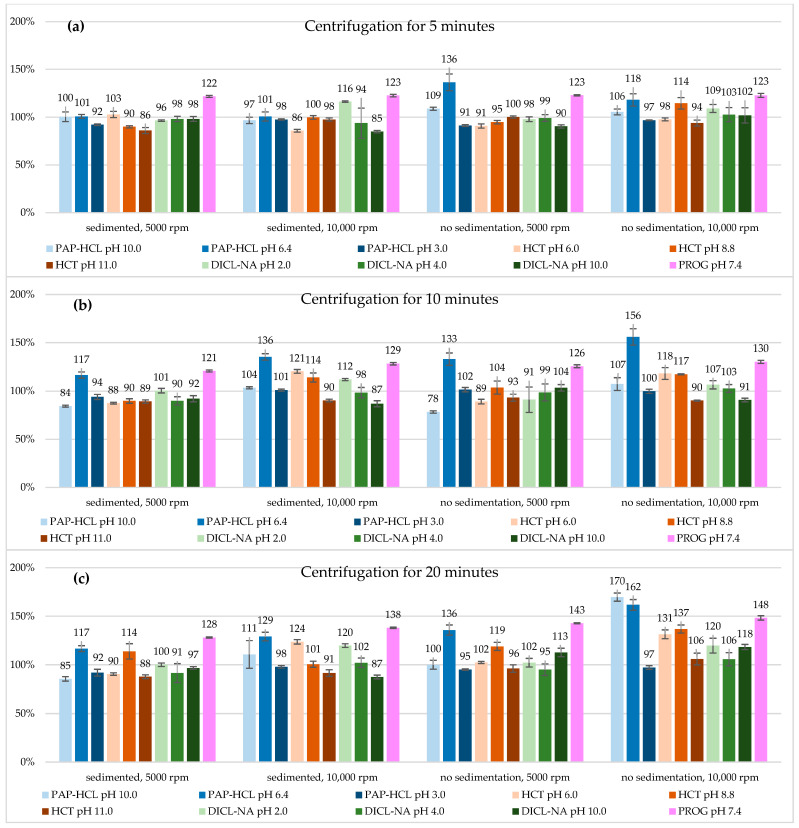
Equilibrium solubility values of the measured compounds (expressed as mean ± SEM), relative to their respective reference value and expressed in %. The duration of centrifugation was (**a**) 5 min, (**b**) 10 min, (**c**) 20 min.

Table 3 presents the solubility values of our compounds after centrifugation. Comparing samples where all centrifugation parameters were the same, skipping the sedimentation step usually resulted in higher solubility values (Figure 3). The reason for this phenomenon is that omitting sedimentation can prevent the system from reaching equilibrium, leading to higher observed solubility values and increased variability. Without adequate sedimentation time, the solid and liquid phases do not fully equilibrate, causing an overestimation of solubility in some samples [18]. Another important thing to mention is that the standard deviation values were in most cases higher in the case of non-sedimented samples, which also supports the fact that these samples have not achieved equilibrium. Therefore, we can conclude that sedimentation should not be skipped when measuring equilibrium solubility and in subsequent parts of our study we will put emphasis on results where sedimentation was used prior to centrifugation.

The speeds of rotation compared were 5000 rpm and 10,000 rpm. Higher centrifugation speed led to increased solubility values in most cases. The most notable case was PAP-HCL at pH 10.0, where after 20 min of centrifugation, the solubility nearly doubled compared to the other samples. Comparing datasets where only the centrifugation speed differed, samples centrifuged at 5000 rpm yielded results closer to the reference value, as seen in Figure 3. This trend is most evident with compounds where a sedimentation step was applied before centrifugation for 5 min. The highest difference between samples centrifuged at 5000 and 10,000 rpm can be observed among the non-sedimented samples, centrifuged for 20 min.

Centrifugation time varied between 5, 10, and 20 min. In most cases, samples centrifuged for 5 min produced the lowest results, with the lowest standard deviation values, therefore it was the most precise method. Comparing the solubility values to the reference, we can also state that the 5 min centrifugation was the most accurate, the closest to 100% (Figure 3). Centrifugation for 10 or 20 min usually resulted in either lower or higher concentrations compared to the reference.

**Figure 4:**
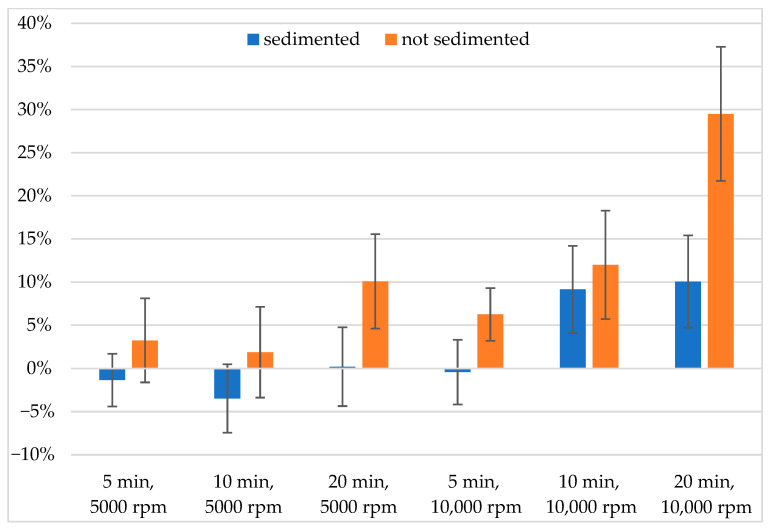
Average deviations from the reference solubility, expressed as %.

To evaluate the effect of all examined parameters, we first calculated the deviations from the reference for all compounds and took their absolute values. Next, for data with the same sets of experimental parameters we calculated the average differences and the standard deviations. These values are displayed in Table 4, showing that the lowest divergence from the reference and the greatest precision can be gained if lower centrifugation speed, time, and a sedimentation step before centrifugation is employed. Longer centrifugation time increased solubility to the largest extent when our samples were not sedimented prior to centrifugation. Figure 4 illustrates the average deviations from the reference grouped by the same sets of parameters, without using absolute values. While some divergences cancel each other out, the most precise and accurate method is still the one with the lowest centrifugation time and rpm, and sedimentation before centrifugation has the most pronounced effect on the measured concentrations of these compounds. In our measurements, combining high centrifugation speed with longer centrifugation time without sedimenting the samples beforehand resulted in the highest increase in measured solubility values.

### 3.3. Comparison of Filtration and Centrifugation

Solubility results obtained through centrifugation were compared with those measured using various filters under the same experimental conditions [16]. Their findings indicate that certain filters can lead to substantial drug loss through adsorption, depending on the compound’s polarity, pH, and the filter material. Table 5 presents the values closest to their respective reference concentrations (expressed as a percentage), along with the range of results for each compound, categorized by pH. This range includes all measurement data obtained for the given API at the specified pH. If the right set of variables are selected (type of filter, centrifugation time and speed), both phase separation methods yield results close to their respective reference values (100%). Choosing a wrong filter can lead to a decrease in measured concentration as high as 50%. In extreme cases the measured concentration dropped to zero, for example, in the case of diclofenac sodium using nylon filter at low pH values.

In contrast, using centrifugation for phase separation, no clear or consistent pH-dependent solubility trends were observed. In all cases, prior sedimentation and centrifugation parameters had a stronger influence on apparent solubility than pH, which showed little impact.

Inaccurate filter selection frequently leads to an underestimation of solubility, while centrifugation may result in overestimation. In case of progesterone, centrifugation caused at least 20% increase in measured solubility. The extremely low solubility of the compound can account for this fact, as even a small increase can cause a larger difference compared to the reference. Centrifugation avoids adsorption losses and is better suited for small-volume or sensitive samples, as it does not require filter saturation. However, under certain conditions (e.g., long spin times, high rpm), unexpectedly high concentrations were observed—especially for papaverine HCl—likely due to re-dissolution of solids.

## 4. Conclusions

Solubility measurements are crucial in drug development, particularly in the early stages where accurate data are essential. In this study, we present an approach that enhances the accuracy of solubility measurements. Phase separation is a key step in these assessments, and even though filtration methods have been widely studied, centrifugation remains relatively underexplored. Our findings contribute to improving measurement precision and support efforts toward interlaboratory standardization. Furthermore, these findings align with the evolving needs of high-throughput screening (HTS) systems, supporting more efficient and standardized solubility testing in modern drug discovery workflows.

While we identified centrifugation conditions that yielded solubility values in good agreement with sedimentation-based results, further investigation revealed that prolonged or overly intense centrifugation can lead to artificially elevated solubility values. This increase in apparent solubility may be attributed to several mechanisms. High centrifugal forces or extended spin durations may cause loosely settled particles to become re-suspended in the supernatant during deceleration or handling. These undissolved solids may remain suspended long enough to be included in the aliquot taken for analysis, increasing the measured solubility. Some compounds tend to form very fine particles or colloidal dispersions that are difficult to fully sediment, even under high centrifugal force. Centrifugation, particularly at high speeds, introduces significant shear forces that may disturb the equilibrium between the solid and dissolved phases. If the system has not fully equilibrated before centrifugation, or if agitation continues during the separation process, solid particles might partially dissolve.

Our final recommendation is thus a sedimentation step before the use of a short (5 min) and slow (5000 rpm) centrifugation method in order to achieve accurate and precise results. If this procedure does not achieve proper phase separation, centrifugation for 10 min at 5000 rpm or 5 min at 10,000 rpm should be the next choice.

The identified optimal centrifugation–sedimentation combinations may not be universally applicable across all compound classes or formulation conditions. In systems containing surfactants or amphiphilic molecules, centrifugation may fail to completely separate micellar or aggregate structures that encapsulate the API. These carriers can retain drug molecules in the supernatant, further inflating the measured concentration and making the interpretation of “free” solubility difficult. Systems with high solvent viscosity present unique challenges as well. In such cases, incomplete phase separation or colloidal retention can lead to significant overestimation of solubility, especially when centrifugation force is insufficient to isolate the dissolved phase effectively. Prior studies [37,43] have shown that higher centrifugal forces may be required in viscous or lipid-based systems to avoid solubility artifacts caused by incomplete settling or aggregate inclusion.

To further establish the applicability of these findings, investigations should be conducted using conditions that closely mimic physiological environments, including relevant pH values, buffer compositions, and biosimilar media, to aid in the high-throughput screening of drug candidates.

## Figures and Tables

**Table 1:** Physico-chemical characteristics of the model compounds [41].

	p*K*_a_	log*P*
PROG	-	3.48
DICL-NA	3.99	4.51
HCT	8.75, 9.96	−0.03
PAP-HCL	6.39	2.95

**Table 2:** Equilibrium solubility (reference) values, obtained after 6 h of stirring and 18 h of sedimentation (standard protocol).

	pH	Solubility (µg/mL)
PROG	7.4	8.98 ± 0.09
DICL-NA	2.0	1.67 ± 0.05
	4.0	2.72 ± 0.16
	10.0	9473 ± 327
HCT	6.0	585 ± 19
	8.8	892 ± 18
	11.0	57,362 ± 85
PAP-HCL	3.0	40,397 ± 930
	6.4	24.2 ± 1.1
	10.0	16.9 ± 1.7

**Table 3:** Equilibrium solubility values, applying centrifugation as phase separation (average ± SEM). *S*_sed_ means the solubility of samples sedimented before centrifugation, while *S*_stir_ denotes the continuously stirred ones, where sedimentation was skipped before centrifugation.

	pH	*S* (µg/mL)	5000 rpm			10,000 rpm		
			5	10	20	5	10	20
PROG	7.4	*S*_sed_	10.9 ± 0.09 *	10.9 ± 0.09 *	11.5 ± 0.06 *	11.0 ± 0.12 *	11.6 ± 0.11 *	12.4 ± 0.06 *
		*S*_stir_	11.0 ± 0.06 *	11.3 ± 0.14 *	12.8 ± 0.05 *	11.0 ± 0.18 *	11.7 ± 0.14 *	13.3 ± 0.20 *
DICL-NA	2.0	*S*_sed_	1.61 ± 0.02	1.68 ± 0.04	1.67 ± 0.03	1.94 ± 0.01	1.87 ± 0.02	2.00 ± 0.03
		*S*_stir_	1.64 ± 0.04	1.52 ± 0.22	1.71 ± 0.07	1.82 ± 0.07	1.78 ± 0.07	2.00 ± 0.13
	4.0	*S*_sed_	2.67 ± 0.08	2.44 ± 0.12	2.49 ± 0.27	2.55 ± 0.06	2.68 ± 0.15	2.78 ± 0.13
		*S*_stir_	2.70 ± 0.10	2.69 ± 0.24	2.58 ± 0.16	2.79 ± 0.20	2.80 ± 0.11	2.88 ± 0.18
	10.0	*S*_sed_	9304 ± 245	8724 ± 304	9164 ± 131	8048 ± 116 *	8236 ± 297	8275 ± 187
		*S*_stir_	8555 ± 161	9812 ± 318	10,678 ± 410	9655 ± 760	8597 ± 176	11,200 ± 251 *
HCT	6.0	*S*_sed_	601.80 ± 18.64	512.21 ± 5.14 *	528.58 ± 7.18	502.08 ± 8.22 *	704.99 ± 11.19 *	723.59 ± 13.80 *
		*S*_stir_	531 ± 12.3	521 ± 14.2	599 ± 6.4	571 ± 9.5	692 ± 35.6 *	769 ± 26.5 *
	8.8	*S*_sed_	803 ± 10.0	801 ± 20.7	1015 ± 70.2	890 ± 17.1	1019 ± 41.6	897.35 ± 28.2
		*S*_stir_	845 ± 16.2	926 ± 60.8	1060 ± 36.9 *	1021 ± 52.8	1048 ± 5.32 *	1221 ± 36.9 *
	11.0	*S*_sed_	49,333 ± 1778	51,165 ± 957	50,270 ± 1099	55,950 ± 980	51,790 ± 786	52,425 ± 1933
		*S*_stir_	57,479 ± 658	53,479 ± 2079	55,100 ± 2240	53,783 ± 1808	51,700 ± 328	60,781 ± 3462
PAP-HCL	3.0	*S*_sed_	37,200 ± 337 *	37,980 ± 1090	37,070 ± 1396 *	39,453 ± 300	40,863 ± 430	39,540 ± 561
		*S*_stir_	36,847 ± 382 *	41,063 ± 922	38,398 ± 279	39,063 ± 197	40,360 ± 905	39,273 ± 777
	6.4	*S*_sed_	24.4 ± 0.48	28.2 ± 0.78	28.2 ± 0.73	24.4 ± 1.17	32.8 ± 0.81 *	31.2 ± 1.08 *
		*S*_stir_	33.0 ± 2.11 *	32.3 ± 1.54 *	32.9 ± 1.26 *	28.6 ± 1.55	37.8 ± 2.07 *	39.1 ± 1.34 *
	10.0	*S*_sed_	17.0 ± 0.86	14.3 ± 0.17	14.4 ± 0.38	16.3 ± 0.57	17.5 ± 0.18	18.7 ± 2.41
		*S*_stir_	18.4 ± 0.28	13.3 ± 0.20	16.9 ± 0.79	17.8 ± 0.50	18.2 ± 1.12	28.7 ± 0.70 *

Values significantly (*p* < 0.05) differing from their respective reference values are presented with a red background and marked with an asterisk.

**Table 4:** The average of the absolute differences from the reference values and their standard deviations.

		Sedimented			Not Sedimented		
		5 min	10 min	20 min	5 min	10 min	20 min
average	5000 rpm	7%	11%	12%	10%	12%	13%
	10,000 rpm	8%	14%	15%	9%	16%	30%
SEM	5000 rpm	0.03	0.04	0.05	0.05	0.05	0.05
	10,000 rpm	0.04	0.05	0.05	0.03	0.06	0.08

**Table 5:** Comparison of filtration and sedimentation.

		Filtration [16]		Centrifugation	
		Closest to Reference (100%)	Result Range	Closest to Reference (100%)	Result Range
papaverine HCl	pH 10.0	95%	62–95%	100%	78–170%
	pH 6.4	95%	42–106%	101%	101–162%
	pH 3.0	95%	85–95%	100%	91–102%
hydrochlorothiazide	pH 6.0	102%	83–103%	98%	86–131%
	pH 8.8	100%	75–100%	100%	90–137%
	pH 11.0	100%	65–119%	100%	86–106%
diclofenac-Na	pH 2.0	93%	0–164%	100%	90–120%
	pH 4.0	103%	0–103%	99%	90–106%
	pH 10.0	99%	64–99%	98%	85–118%
progesterone	pH 7.4	98%	55–98%	121%	121–148%

## Data Availability

The original contributions presented in this study are included in the article. Further inquiries can be directed to the corresponding author.

## Supplementary Materials

The following supporting information can be downloaded at: https://www.mdpi.com/article/10.3390/mps8050116/s1.

## Abbreviations

The following abbreviations are used in this manuscript:

SSF	Saturation shake-flask
API	Active pharmaceutical ingredient
BCS	Biopharmaceutics classification system
PROG	Progesterone
DICL-NA	Diclofenac sodium
HCT	Hydrochlorothiazide
PAP-HCL	Papaverine hydrochloride
*S*_sed_	Solubility of samples sedimented before centrifugation
*S*_stir_	Solubility of samples continuously stirred before centrifugation
