# Supplementary material for: Effects of Different Centrifugation Parameters on Equilibrium Solubility Measurements
Source: Methods Protoc. 2025 Oct 2;8(5):116. doi: 10.3390/mps8050116 (PMC12566593; doi:10.3390/mps8050116)
Supplement: Supplementary file 1 [file mps-08-00116-s001.zip › mps-3835587-supplementary.pdf]

## Supplementary Materials

**Q-Q plot**

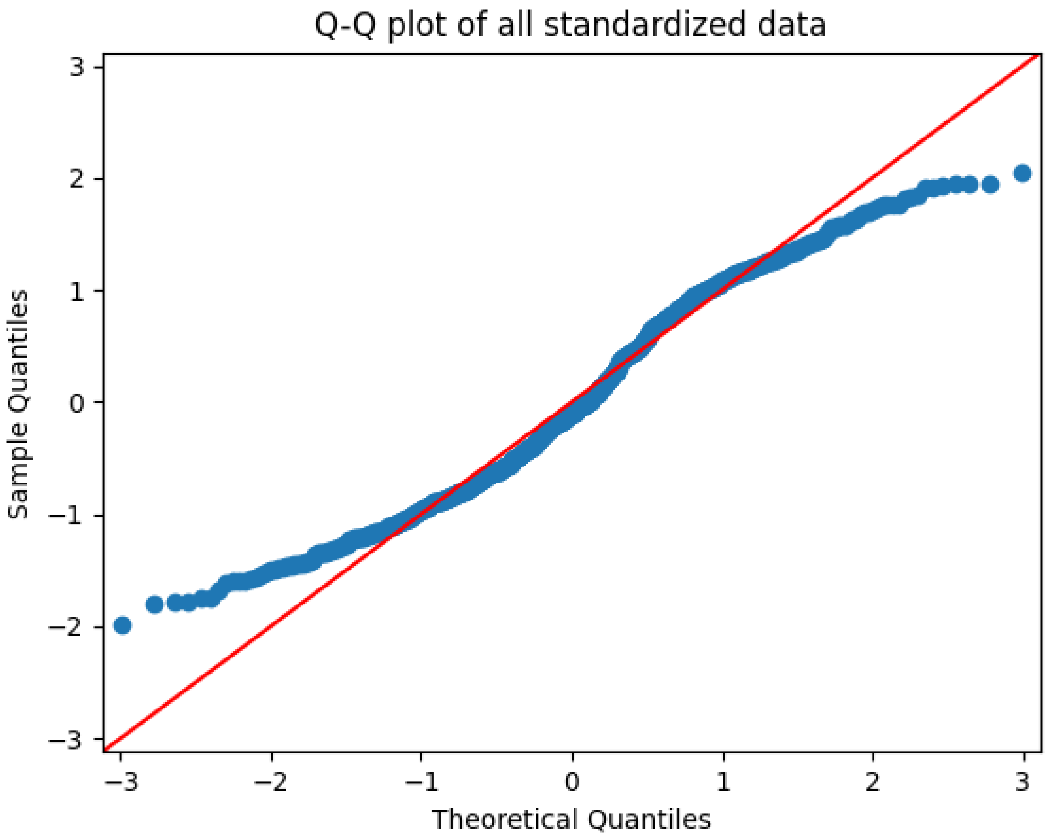


**Figure S1.** Q–Q plot of standardized solubility values across all groups. The plot was used to visually assess the normality of the data prior to statistical analysis.

**Table S1.** Number of measurements for each combination of parameters.

| | | | sedimented | | | not sedimented | | |
|---|---|---|---|---|---|---|---|---|
| | | | 5 min | 10 min | 20 min | 5 min | 10 min | 20 min |
| papaverine-HCl | pH 10.0 | 5000 rpm | 3 | 6 | 6 | 5 | 4 | 6 |
| | | 10000 rpm | 4 | 5 | 5 | 4 | 5 | 5 |
| | pH 6.4 | 5000 rpm | 6 | 3 | 5 | 3 | 4 | 6 |
| | | 10000 rpm | 4 | 5 | 6 | 6 | 4 | 5 |
| | pH 3.0 | 5000 rpm | 3 | 5 | 4 | 3 | 3 | 6 |
| | | 10000 rpm | 4 | 3 | 3 | 4 | 4 | 4 |
| hydrochlorothia-zide | pH 6.0 | 5000 rpm | 6 | 6 | 6 | 6 | 6 | 6 |
| | | 10000 rpm | 6 | 6 | 6 | 6 | 6 | 6 |
| | pH 8.8 | 5000 rpm | 6 | 6 | 6 | 6 | 6 | 6 |
| | | 10000 rpm | 6 | 6 | 6 | 6 | 6 | 6 |
| | pH 11.0 | 5000 rpm | 3 | 5 | 5 | 7 | 7 | 7 |
| | | 10000 rpm | 5 | 5 | 6 | 6 | 6 | 8 |
| diclofenac-Na | pH 2.0 | 5000 rpm | 3 | 8 | 6 | 5 | 4 | 8 |
| | | 10000 rpm | 4 | 5 | 7 | 5 | 6 | 7 |
| | pH 4.0 | 5000 rpm | 7 | 6 | 6 | 4 | 6 | 5 |
| | | 10000 rpm | 5 | 6 | 7 | 5 | 5 | 5 |
| | pH 10.0 | 5000 rpm | 6 | 7 | 6 | 6 | 6 | 6 |
| | | 10000 rpm | 6 | 6 | 6 | 6 | 6 | 6 |
| progesterone | pH 7.4 | 5000 rpm | 6 | 8 | 8 | 8 | 8 | 8 |
| | | 10000 rpm | 8 | 8 | 8 | 8 | 8 | 8 |

**Table S2.** Buffer and API amount for each pH.

| | | Buffer amount (mL) | API amount |
|---|---|---|---|
| papaverine-HCl | pH 10.0 | 20 mL | ~ 0.006 g |
| | pH 6.4 | 15 mL | ~ 0.025 g |
| | pH 3.0 | 15 mL | ~ 0.570 g |
| hydrochlorothiazide | pH 6.0 | 15 mL | ~ 0.060 g |
| | pH 8.8 | 15 mL | ~ 0.450 g |
| | pH 11.0 | 5 mL | ~ 1.350 g |
| diclofenac-Na | pH 2.0 | 20 mL | ~ 0.002 g |
| | pH 4.0 | 20 mL | ~ 0.002 g |
| | pH 10.0 | 15 mL | ~ 0.200 g |
| progesterone | pH 7.4 | 20 mL | ~ 0.006 g |

**Table S3.** Calibration parameters.

| | | $R^2$ | **λ 2nd derivate** |
|---|---|---|---|
| papaverine-HCl | pH 10.0 | 0.9997 | 292-306 nm |
| | | 0.9997 | 292-306 nm |
| | | 0.9996 | 292-306 nm |
| | pH 6.4 | 0.9985 | 292-306 nm |
| | | 0.9991 | 292-306 nm |
| | | 0.9988 | 292-306 nm |
| | pH 3.0 | 0.9992 | 292-306 nm |
| | | 0.9993 | 292-306 nm |
| | | 0.9993 | 292-306 nm |
| hydrochlorothiazide | pH 6.0 | 0.9998 | 286-300 nm |
| | | 0.9996 | 286-300 nm |
| | | 0.9997 | 286-300 nm |
| | pH 8.8 | 0.9990 | 286-300 nm |
| | | 0.9998 | 286-300 nm |
| | | 0.9989 | 286-300 nm |
| | pH 11.0 | 0.9995 | 286-300 nm |
| | | 0.9998 | 286-300 nm |
| | | 0.9999 | 286-300 nm |
| diclofenac-Na | pH 2.0 | 0.9980 | 300-320 nm |
| | | 0.9993 | 300-320 nm |
| | | 0.9985 | 300-320 nm |
| | pH 4.0 | 0.9991 | 300-320 nm |
| | | 0.9996 | 300-320 nm |
| | | 0.9997 | 300-320 nm |
| | pH 10.0 | 0.9999 | 300-320 nm |
| | | 0.9999 | 300-320 nm |
| | | 0.9999 | 300-320 nm |
| progesterone | pH 7.4 | 0.9998 | 268-280 nm |
| | | 0.9998 | 268-280 nm |
| | | 0.9998 | 268-280 nm |

**Dunnet's ANOVA**

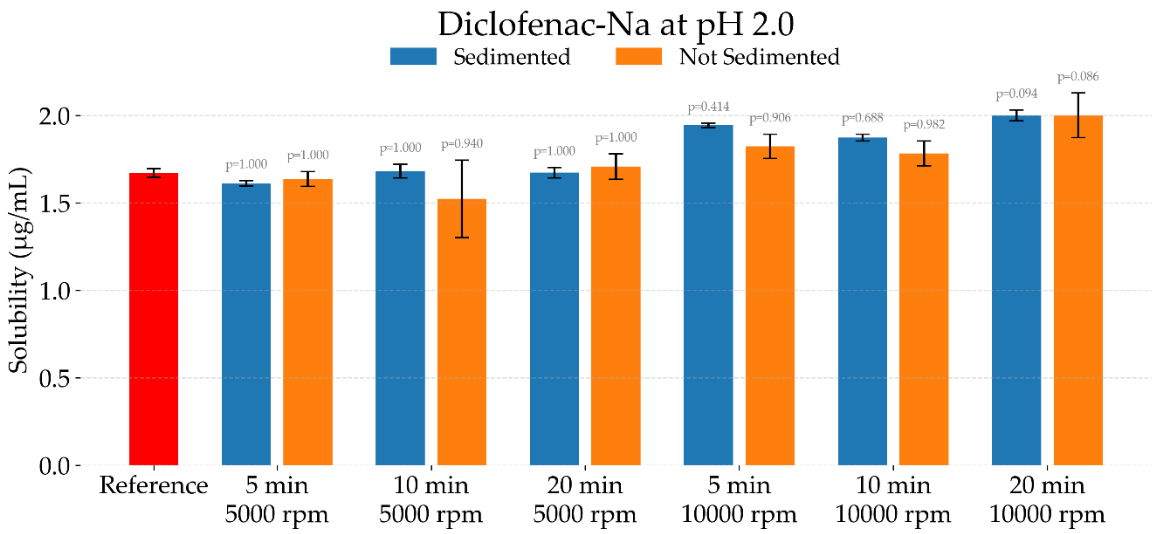


**Figure S2.** Solubility of Diclofenac-Na under different centrifugation protocols at pH 2.0. Bars represent mean ± SEM. Significant differences from the reference condition were determined using one-way ANOVA with Dunnett's post-hoc test ($p < 0.05$). Exact p-values and significant differences are indicated by asterisks above the bars.

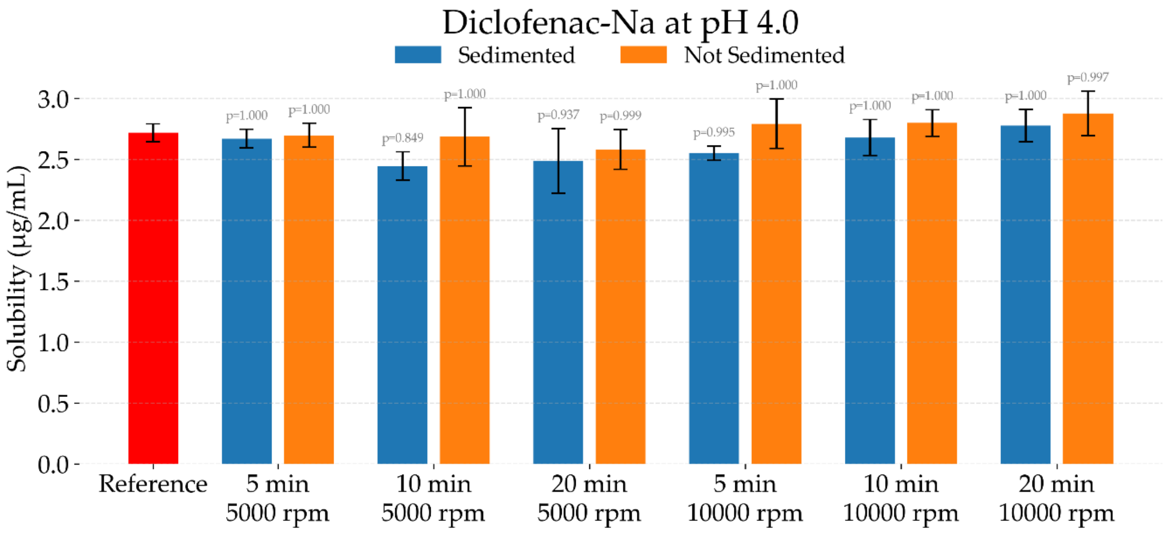


**Figure S3.** Solubility of Diclofenac-Na under different centrifugation protocols at pH 4.0. Bars represent mean ± SEM. Significant differences from the reference condition were determined using one-way ANOVA with Dunnett's post-hoc test ($p < 0.05$). Exact p-values and significant differences are indicated by asterisks above the bars.

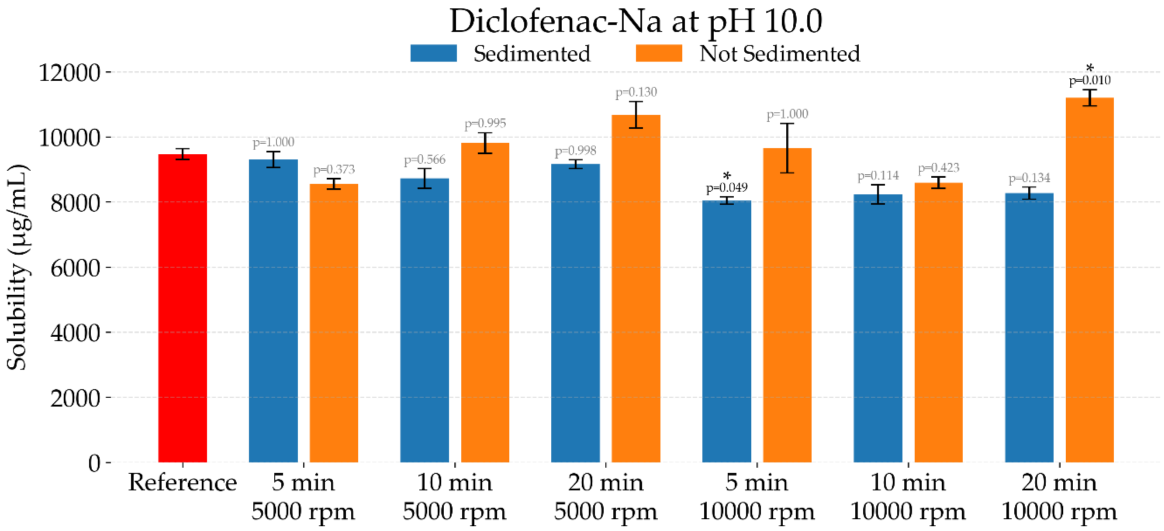


**Figure S4.** Solubility of Diclofenac-Na under different centrifugation protocols at pH 10.0. Bars represent mean ± SEM. Significant differences from the reference condition were determined using one-way ANOVA with Dunnett's post-hoc test ($p < 0.05$). Exact p-values and significant differences are indicated by asterisks above the bars.

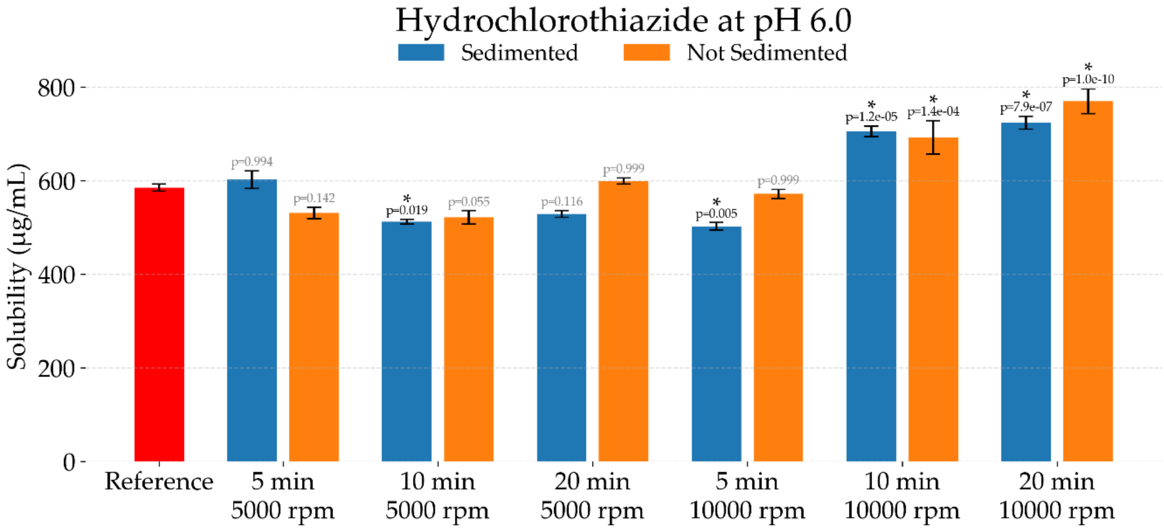


**Figure S5.** Solubility of Hydrochlorothiazide under different centrifugation protocols at pH 6.0. Bars represent mean ± SEM. Significant differences from the reference condition were determined using one-way ANOVA with Dunnett's post-hoc test ($p < 0.05$). Exact p-values and significant differences are indicated by asterisks above the bars.

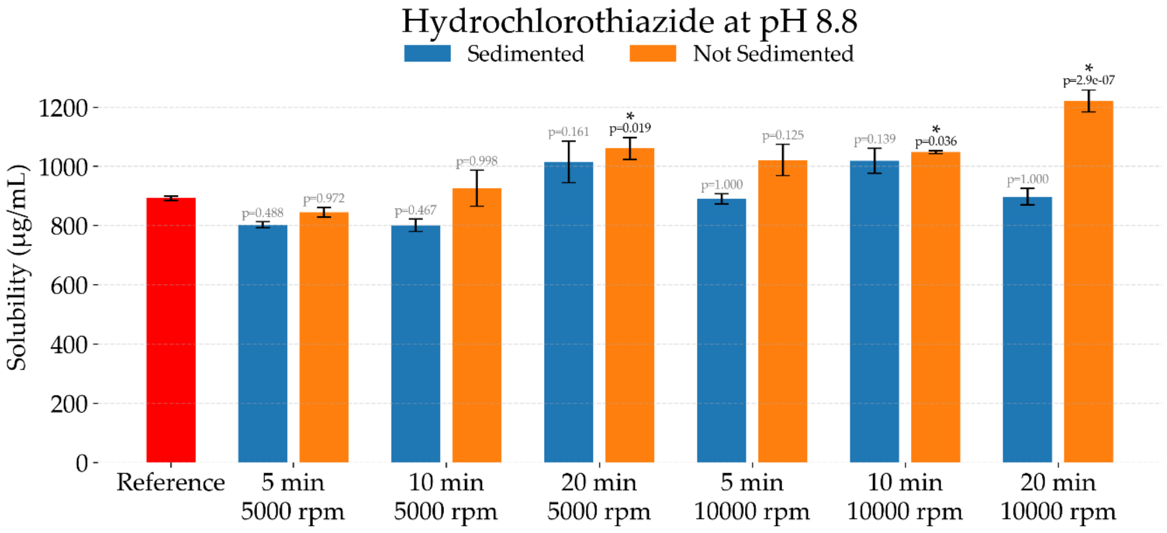


**Figure S6.** Solubility of Hydrochlorothiazide under different centrifugation protocols at pH 8.8. Bars represent mean ± SEM. Significant differences from the reference condition were determined using one-way ANOVA with Dunnett's post-hoc test ($p < 0.05$). Exact p-values and significant differences are indicated by asterisks above the bars.

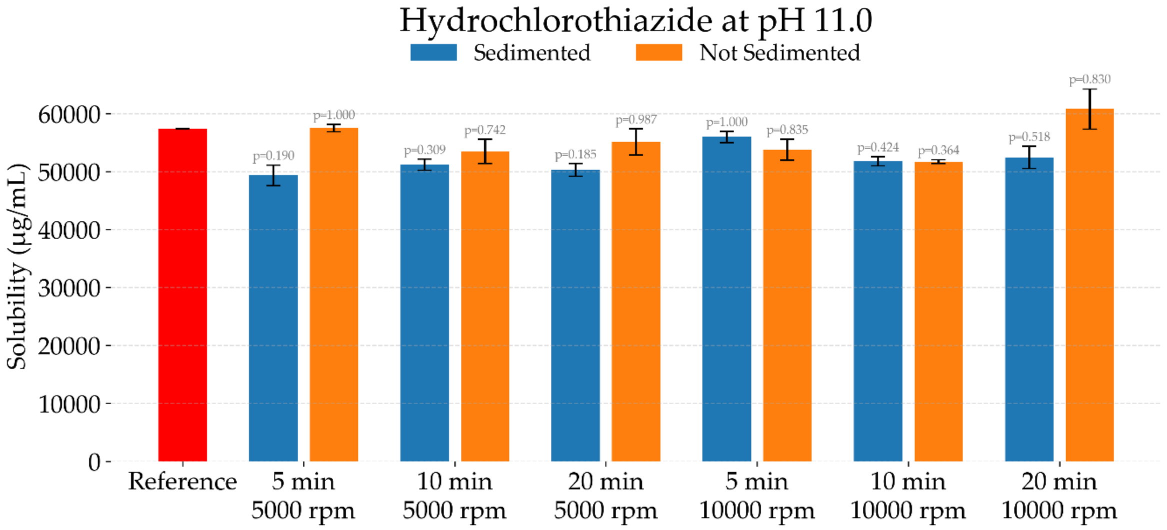


**Figure S7.** Solubility of Hydrochlorothiazide under different centrifugation protocols at pH 11.0. Bars represent mean ± SEM. Significant differences from the reference condition were determined using one-way ANOVA with Dunnett's post-hoc test ($p < 0.05$). Exact p-values and significant differences are indicated by asterisks above the bars.

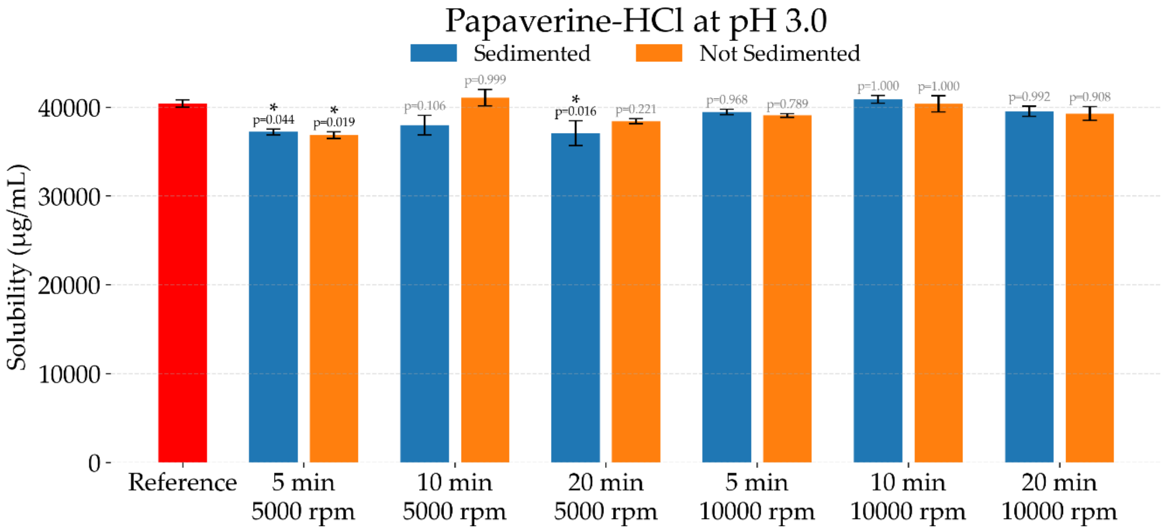


**Figure S8.** Solubility of Papaverine-HCl under different centrifugation protocols at pH 3.0. Bars represent mean ± SEM. Significant differences from the reference condition were determined using one-way ANOVA with Dunnett's post-hoc test ($p < 0.05$). Exact p-values and significant differences are indicated by asterisks above the bars.

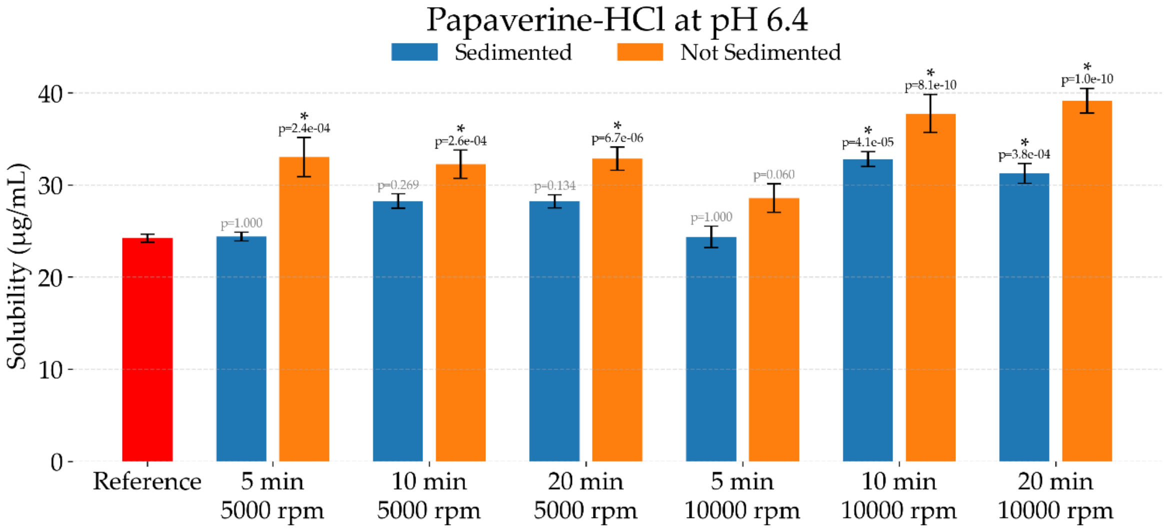


**Figure S9.** Solubility of Papaverine-HCl under different centrifugation protocols at pH 6.4. Bars represent mean ± SEM. Significant differences from the reference condition were determined using one-way ANOVA with Dunnett's post-hoc test ($p < 0.05$). Exact p-values and significant differences are indicated by asterisks above the bars.

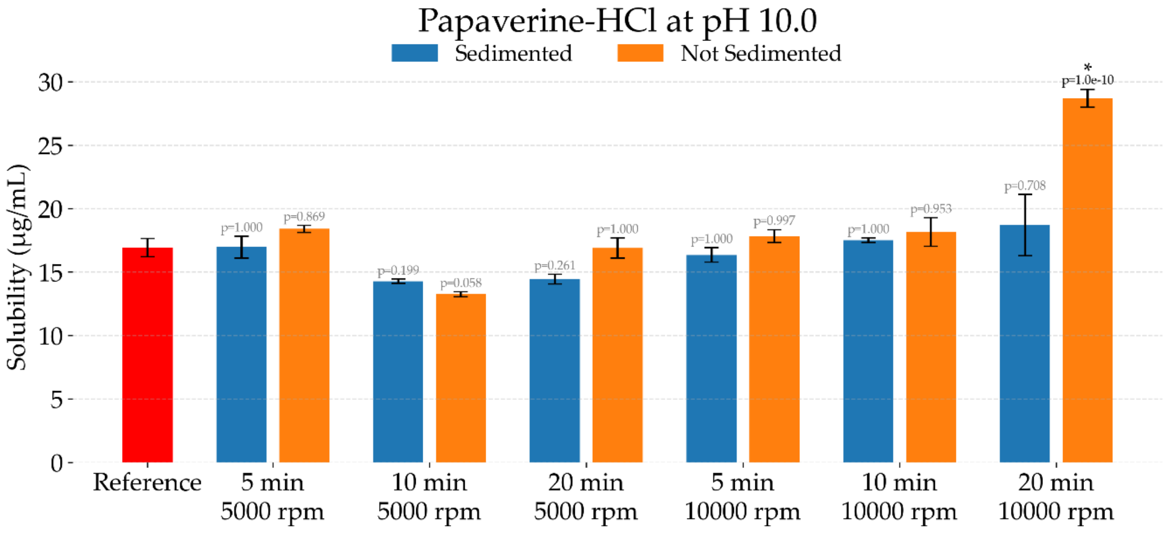


**Figure S10.** Solubility of Papaverine-HCl under different centrifugation protocols at pH 10.0. Bars represent mean ± SEM. Significant differences from the reference condition were determined using one-way ANOVA with Dunnett's post-hoc test ($p < 0.05$). Exact p-values and significant differences are indicated by asterisks above the bars.

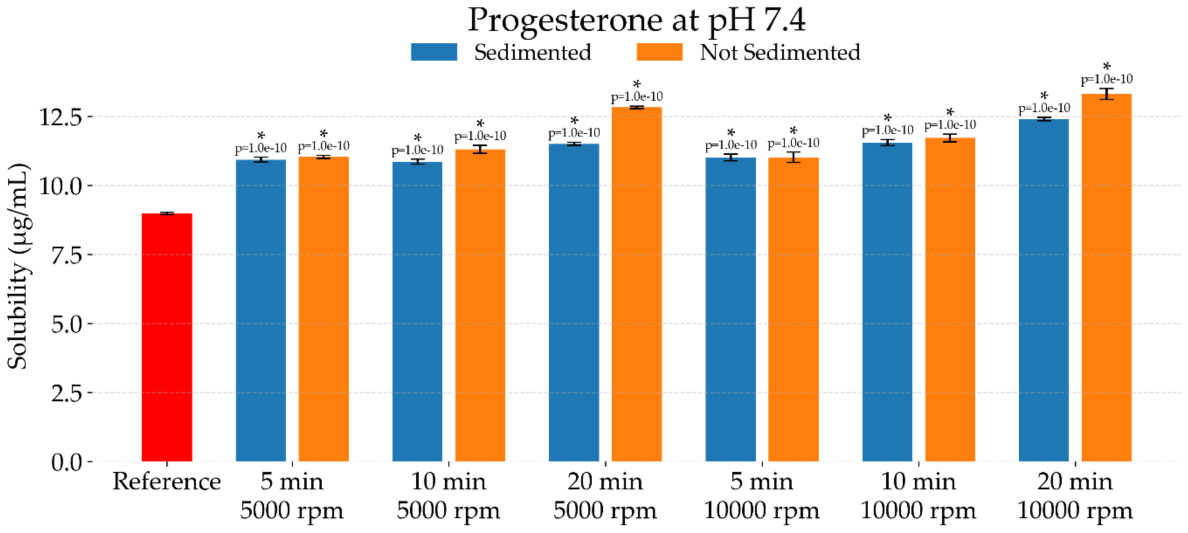


**Figure S11.** Solubility of Progesterone under different centrifugation protocols at pH 7.4. Bars represent mean ± SEM. Significant differences from the reference condition were determined using one-way ANOVA with Dunnett's post-hoc test ($p < 0.05$). Exact p-values and significant differences are indicated by asterisks above the bars.

**Welch's t-tests**

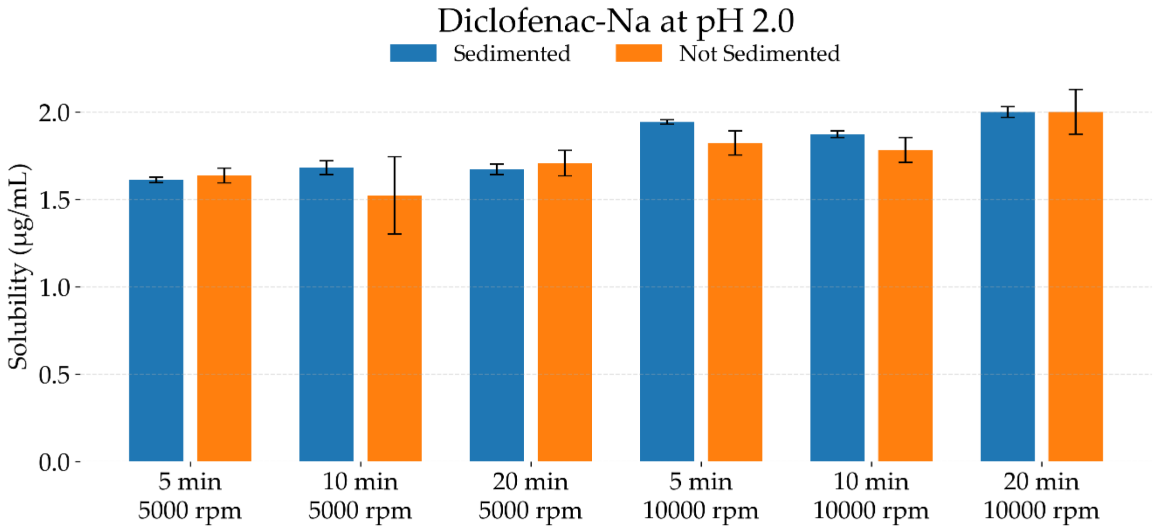


**Figure S12.** Solubility of Diclofenac-Na under a specific centrifugation protocol at pH 2.0. Bars represent mean ± SEM. Significant differences between sedimented and non-sedimented samples were evaluated using Welch's unpaired two-tailed t-test, with significance thresholds of $p < 0.05$ (*), 0.01(**), and 0.001 (***). Significant differences, if any, are indicated by asterisks above the bars.

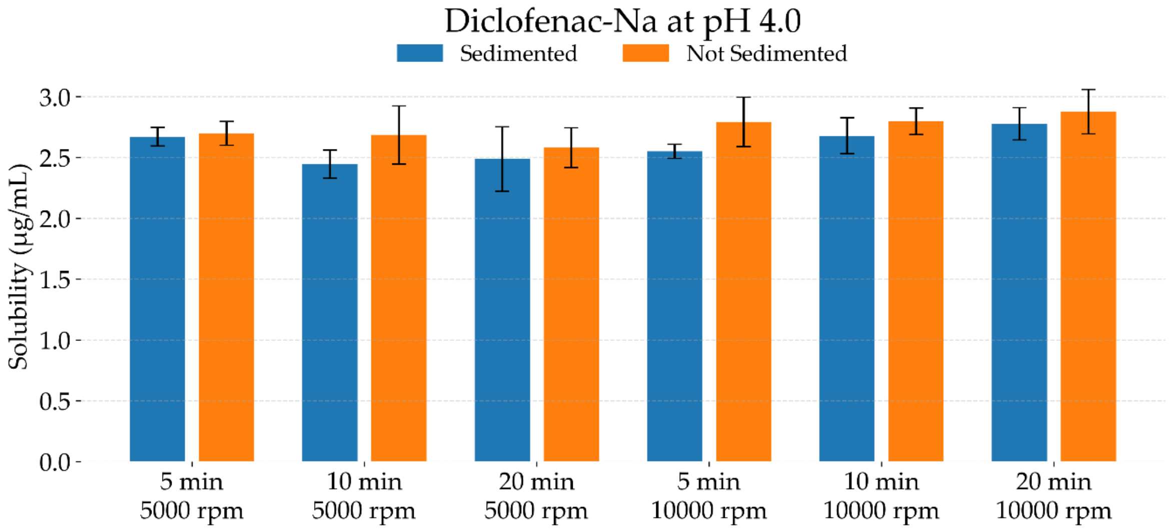


**Figure S13.** Solubility of Diclofenac-Na under a specific centrifugation protocol at pH 4.0. Bars represent mean ± SEM. Significant differences between sedimented and non-sedimented samples were evaluated using Welch's unpaired two-tailed t-test, with significance thresholds of $p < 0.05$ (*), 0.01(**), and 0.001 (***). Significant differences, if any, are indicated by asterisks above the bars.

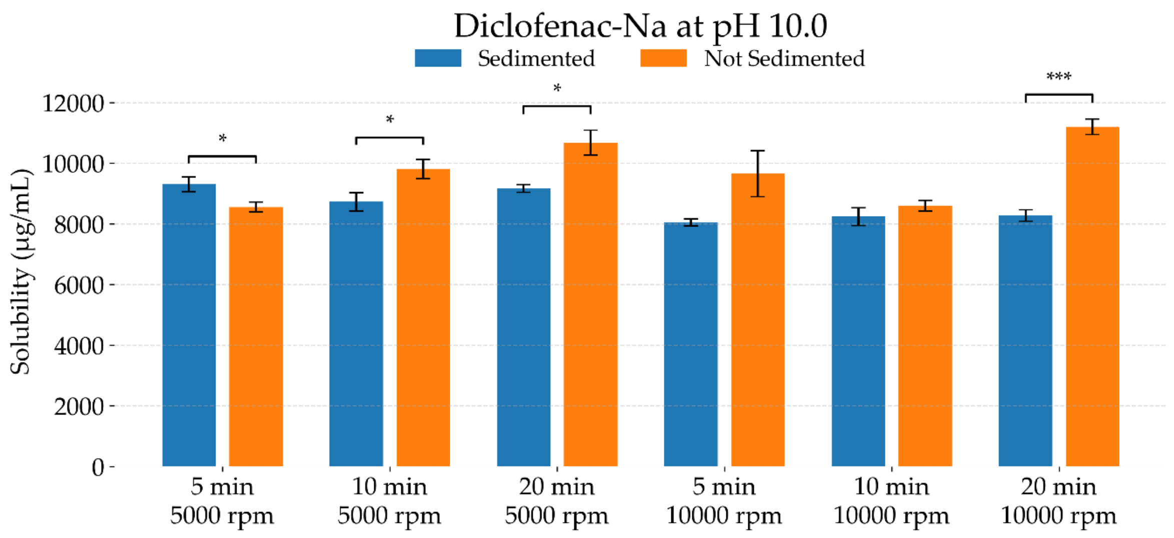


**Figure S14.** Solubility of Diclofenac-Na under a specific centrifugation protocol at pH 10.0. Bars represent mean ± SEM. Significant differences between sedimented and non-sedimented samples were evaluated using Welch's unpaired two-tailed t-test, with significance thresholds of $p < 0.05$ (*), 0.01(**), and 0.001 (***). Significant differences, if any, are indicated by asterisks above the bars.

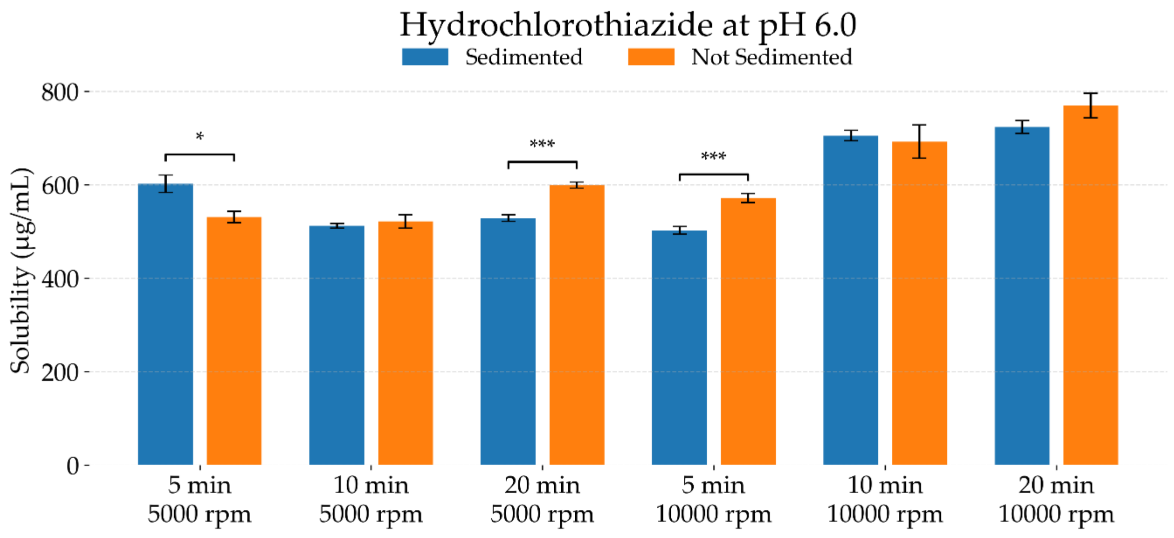


**Figure S15.** Solubility of Hydrochlorothiazide under a specific centrifugation protocol at pH 6.0. Bars represent mean ± SEM. Significant differences between sedimented and non-sedimented samples were evaluated using Welch's unpaired two-tailed t-test, with significance thresholds of $p < 0.05$ (*), 0.01(**), and 0.001 (***). Significant differences, if any, are indicated by asterisks above the bars.

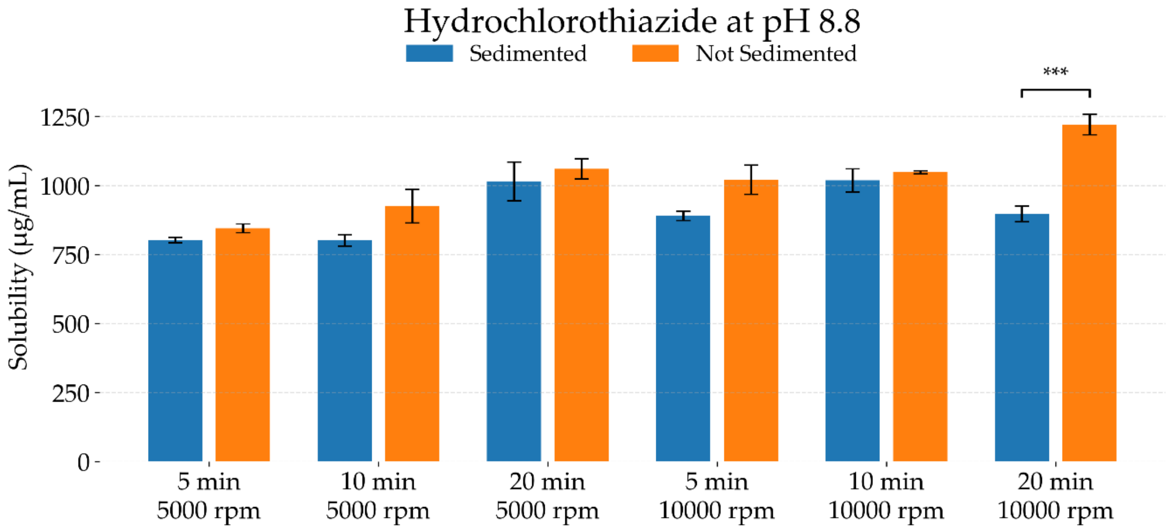


**Figure S16.** Solubility of Hydrochlorothiazide under a specific centrifugation protocol at pH 8.8. Bars represent mean ± SEM. Significant differences between sedimented and non-sedimented samples were evaluated using Welch's unpaired two-tailed t-test, with significance thresholds of $p < 0.05$ (*), 0.01(**), and 0.001 (***). Significant differences, if any, are indicated by asterisks above the bars.

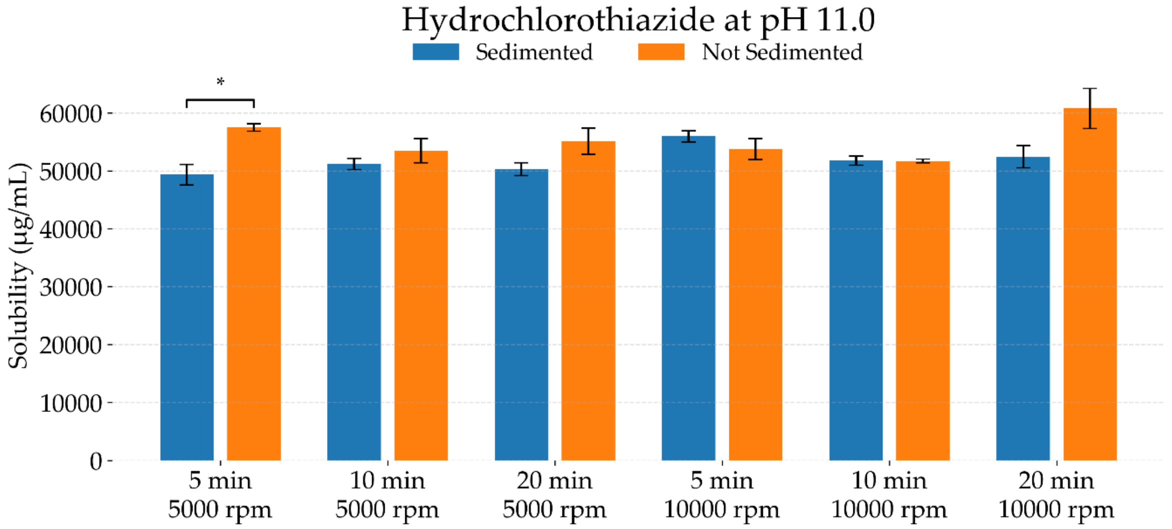


**Figure S17.** Solubility of Hydrochlorothiazide under a specific centrifugation protocol at pH 11.0. Bars represent mean ± SEM. Significant differences between sedimented and non-sedimented samples were evaluated using Welch's unpaired two-tailed t-test, with significance thresholds of $p < 0.05$ (*), 0.01(**), and 0.001 (***). Significant differences, if any, are indicated by asterisks above the bars.

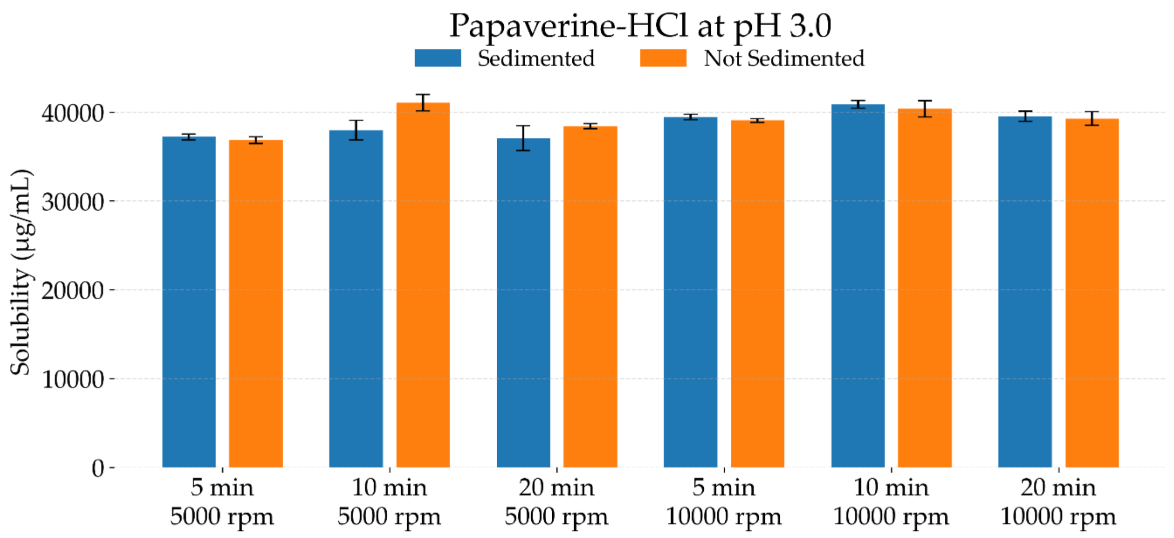


**Figure S18.** Solubility of Papaverine-HCl under a specific centrifugation protocol at pH 3.0. Bars represent mean ± SEM. Significant differences between sedimented and non-sedimented samples were evaluated using Welch's unpaired two-tailed t-test, with significance thresholds of $p < 0.05$ (*), 0.01(**), and 0.001 (***). Significant differences, if any, are indicated by asterisks above the bars.

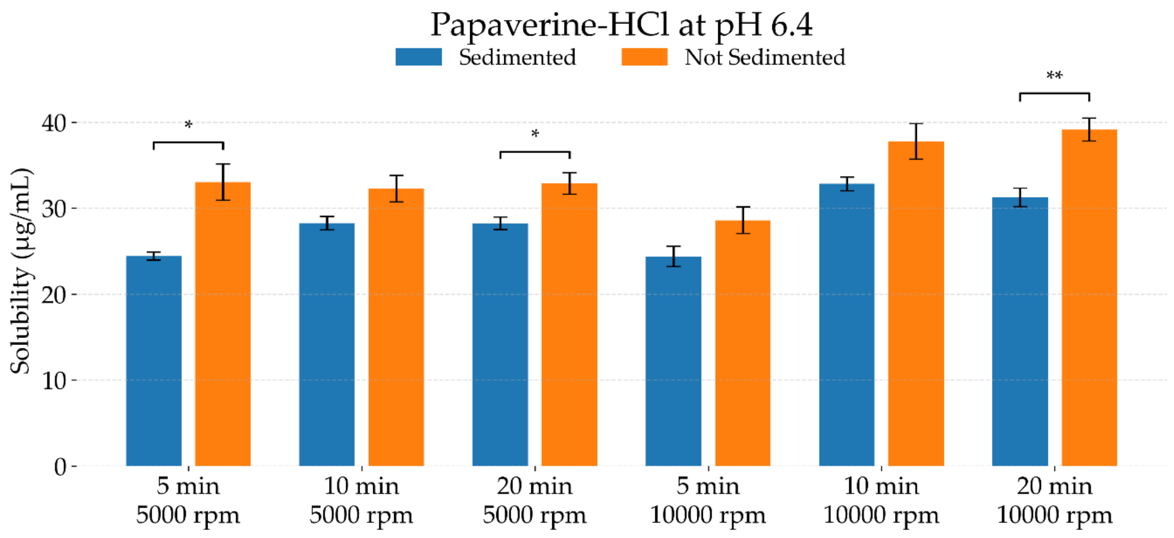


**Figure S19.** Solubility of Papaverine-HCl under a specific centrifugation protocol at pH 6.4. Bars represent mean ± SEM. Significant differences between sedimented and non-sedimented samples were evaluated using Welch's unpaired two-tailed t-test, with significance thresholds of $p < 0.05$ (*), 0.01(**), and 0.001 (***). Significant differences, if any, are indicated by asterisks above the bars.

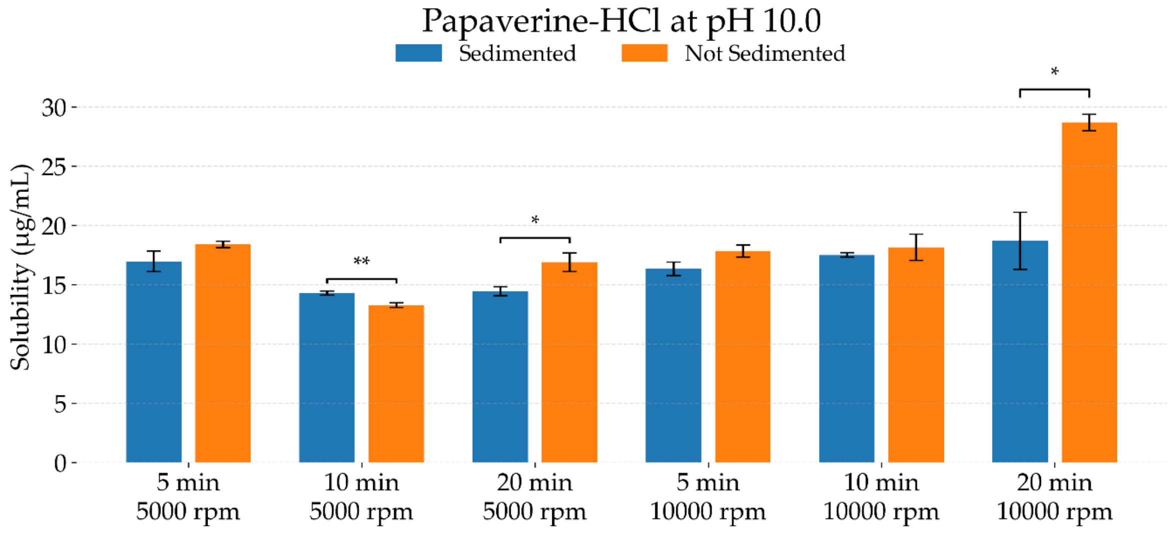


**Figure S20.** Solubility of Papaverine-HCl under a specific centrifugation protocol at pH 10.0. Bars represent mean ± SEM. Significant differences between sedimented and non-sedimented samples were evaluated using Welch's unpaired two-tailed t-test, with significance thresholds of $p < 0.05$ (*), 0.01(**), and 0.001 (***). Significant differences, if any, are indicated by asterisks above the bars.

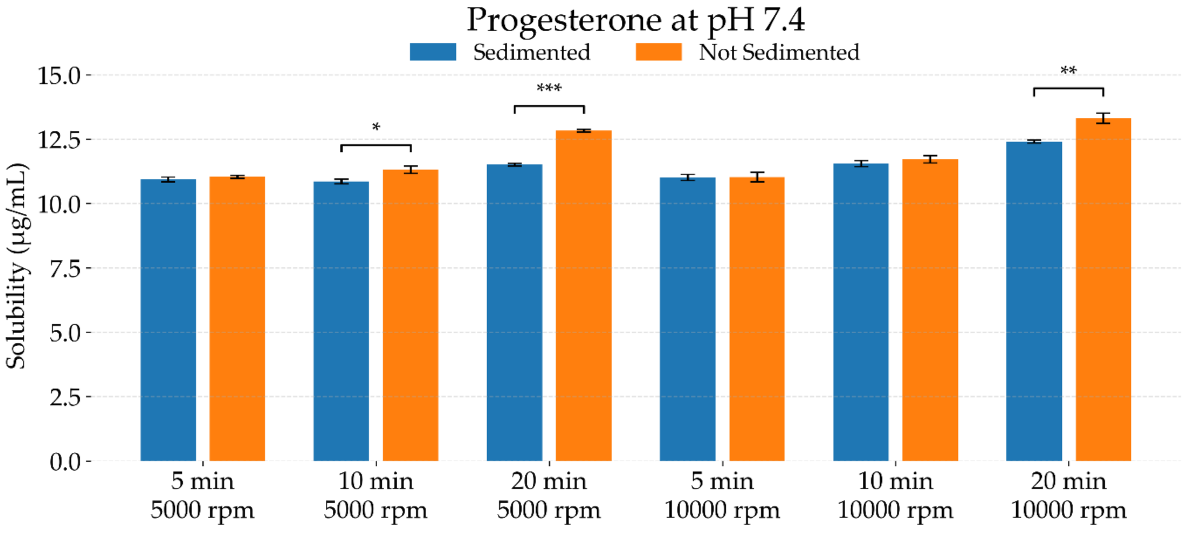


**Figure S21.** Solubility of Progesterone under a specific centrifugation protocol at pH 7.4. Bars represent mean ± SEM. Significant differences between sedimented and non-sedimented samples were evaluated using Welch's unpaired two-tailed t-test, with significance thresholds of $p < 0.05$ (*), 0.01(**), and 0.001 (***). Significant differences, if any, are indicated by asterisks above the bars.
